# Tuning Corn Zein-Chitosan Biocomposites via Mild Alkaline Treatment: Structural and Physicochemical Property Insights

**DOI:** 10.3390/polym17152161

**Published:** 2025-08-07

**Authors:** Nagireddy Poluri, Creston Singer, David Salas-de la Cruz, Xiao Hu

**Affiliations:** 1 Department of Physics and Astronomy, Rowan University, Glassboro, NJ 08028, USA; poluri34@students.rowan.edu; 2 Department of Chemistry and Biochemistry, Rowan University, Glassboro, NJ 08028, USA; 3 Center for Computational and Integrative Biology, Rutgers University, Camden, NJ 08102, USA; cas701@scarletmail.rutgers.edu (C.S.); ds1191@camden.rutgers.edu (D.S.-d.l.C.); 4 Department of Chemistry, Rutgers University, Camden, NJ 08102, USA; 5 Department of Biological and Biomedical Sciences, Rowan University, Glassboro, NJ 08028, USA

**Keywords:** zein, chitosan, composite film, NaOH treatment, secondary structure, hydrogen bonding

## Abstract

This study investigates the structural and functional enhancement of corn zein–chitosan composites via mild alkaline treatment to develop biodegradable protein-polysaccharide materials for diverse applications. Films with varying zein-to-chitosan ratios were fabricated and characterized using Fourier-transform infrared spectroscopy (FTIR), thermogravimetric analysis (TGA), differential scanning calorimetry (DSC), and scanning electron microscopy (SEM). Both untreated and sodium hydroxide (NaOH)-treated films were evaluated to assess changes in physicochemical properties. FTIR analysis revealed that NaOH treatment promoted deprotonation of chitosan’s amine groups, partial removal of ionic residues, and increased deacetylation, collectively enhancing hydrogen bonding and resulting in a denser molecular network. Simultaneously, partial unfolding of zein’s α-helical structures improved conformational flexibility and strengthened interactions with chitosan. These molecular-level changes led to improved thermal stability, reduced degradation, and the development of porous microstructures. Controlled NaOH treatment thus provides an effective strategy to tailor the physicochemical properties of zein–chitosan composite films, supporting their potential in sustainable food packaging, wound healing, and drug delivery applications.

## 1. Introduction

Biopolymer-based composite materials have emerged as promising candidates for advanced biomedical applications due to their inherent biocompatibility, biodegradability, and capacity for molecular customization. These materials can be engineered to exhibit a broad range of physical and mechanical properties, such as elasticity, toughness, and structural integrity, by strategically optimizing molecular-level interactions. Natural structural proteins, including collagen, silk, zein, and elastin, have evolved over thousands of years to fulfill specialized biological functions; however, their individual limitations often necessitate further enhancement for broader applicability. Although recombinant approaches allow for precise molecular design, their scalability remains a significant challenge [1]. Blending distinct biopolymers offers a practical strategy to overcome these limitations by combining complementary properties and improving processability and material uniformity. The development of multifunctional protein-polysaccharide-based composites through such blending strategies provides a versatile and scalable platform to tailor material properties for targeted in vitro and in vivo applications, including drug delivery, biosensing, and tissue engineering. The exceptional functionality of natural biomaterials arises from their highly ordered molecular architectures, such as β-sheets, random coils, and triple helices, which are governed by specific amino acid sequences and give rise to a wide range of tunable physical, chemical, and biological properties [2].

Zein is a class of alcohol-soluble storage proteins derived from corn, categorized into four subclasses α, β, γ, and δ based on their solubility profiles and amino acid sequences. Among these, α-zein predominates, constituting approximately 70–85% of the total zein fraction, with γ-zein representing 10–20%. Zein proteins are characterized by a high content of hydrophobic amino acids such as leucine and proline, which contribute to their distinct physicochemical properties. All zein fractions are rich in hydrophobic and neutral amino acids such as leucine, proline, and alanine, while also containing some polar residues like glutamine. α-Zein is composed of highly similar repetitive sequences and is characterized by a high content of α-helices. Unlike many other proteins, zein notably lacks lysine and tryptophan and has only small amounts of arginine and histidine [3,4,5]. These distinctive amino acid compositions contribute to zein’s unique solubility profile, which is primarily limited to solvents like acetone, acetic acid, formic acid, aqueous alcohols, and alkaline solutions [3,6]. Zeins can be fabricated in many forms, such as nanoparticles [7], nanofibers [8,9], films [6,9], or coatings [10] for various material applications. Despite its useful properties, zein typically exhibits low mechanical strength. Studies have shown that this limitation can be addressed through crosslinking techniques and by forming composites with mechanically robust natural polymers, both of which have demonstrated potential in improving its structural performance [11,12,13].

Chitosan, a naturally derived linear polysaccharide obtained from the deacetylation of chitin, is a biodegradable, biocompatible, non-toxic, and renewable polymer [14]. Composed of D-glucosamine and N-acetyl-D-glucosamine units linked by β-(1→4) glycosidic bonds [15], chitosan can be readily modified and is known for its strong capacity to interact with metal ions and enzymes [16,17]. Beyond its widespread use in food and agriculture, chitosan is also valued for its film-forming capabilities [16]. A key distinction between chitin and chitosan lies in their solubility: chitosan is soluble in acidic aqueous solutions such as phosphoric acid [16], acetic acid [18], and formic acid [19], as well as in ionic liquids [20] and even water [21], whereas chitin is not [16]. Chitosan typically exhibits an intrinsic cationic nature, which is rare among naturally occurring polymers and plays a critical role in its functional versatility. This positive charge enables strong electrostatic interactions with negatively charged biomolecules, including proteins, enzymes, cell membranes, and various anionic polymers. Such interactions are fundamental to its effectiveness in a wide range of applications, including targeted drug delivery, gene transfection, tissue engineering, and the development of responsive biomaterials [22]. Recent studies have also highlighted the growing versatility of chitosan-based composites in environmental, biomedical, and food-related applications. For example, chitosan crosslinked with epichlorohydrin and blended with activated carbon has demonstrated enhanced CO_2_ adsorption efficiency, optimized using statistical and machine learning models [23]. Dual-layer PVA/chitosan films reinforced with natamycin and boric acid have been successfully fabricated using 3D printing and electrospinning techniques for various in vitro applications [24]. Additionally, crosslinked chitosan-based oleogels have shown improved mechanical properties, making them promising alternatives to conventional solid fats in food systems [25]. These advancements underscore chitosan’s adaptability and support its continued exploration in multifunctional composite materials.

Natural protein materials such as zein and polysaccharides like chitosan possess unique functional properties. When combined, these biopolymers can form composites tailored for specific applications. In this study, zein–chitosan composites with three different blend ratios, along with pure zein and chitosan control films, were blended to systematically investigate their structural, thermal, and morphological characteristics. The samples were subsequently treated with sodium hydroxide (NaOH) to assess the impact of alkaline conditions on material properties. Alkaline treatment is known to influence the degree of chitosan deacetylation, alter interactions between biopolymer chains, and modify the overall composite structure [16]. Furthermore, mild alkaline treatment with sodium hydroxide is widely employed to improve the water stability of silk–chitosan composites, a critical property for their successful application in biomedical fields [19]. Recent work by Yu et al. [26] demonstrated that NaOH/urea neutralization of chitosan/carboxymethylcellulose films enhanced film cohesion, reduced surface deprotonation, and improved mechanical strength and barrier properties. In a similar investigation, the addition of NaOH (up to 0.2 M) during the extrusion of starch–zein composite films significantly reduced zein aggregation, improved interfacial compatibility, and enhanced elongation at break by ~28%, as supported by FTIR evidence indicating increased molecular mobility and improved dispersion [27]. Building on these findings, we aimed to systematically investigate the effect of mild alkaline treatment on the molecular interactions and structural rearrangements within zein–chitosan composite films. NaOH-induced enhancements improved zein–chitosan materials’ structural integrity, thermal stability, and surface morphology—factors critical for determining their suitability for biomedical applications. These findings highlight how the combined functional attributes of zein and chitosan, together with controlled post-treatment, can be leveraged to develop composites with tunable properties for advanced biomedical use.

## 2. Materials and Methods

### 2.1. Preparation of Materials

Zein protein powder, with a purity exceeding 87%, was kindly provided by POET, LLC (Sioux Falls, SD, USA). Analytical grade formic acid (98%) was obtained from EMD Millipore Corporation (Burlington, MA, USA). Low molecular weight chitosan, with an average viscometric molecular weight of 50–190 kDa and approximately 75% deacetylation, along with sodium hydroxide (NaOH), was purchased from Sigma-Aldrich (St. Louis, MO, USA). All materials were used as received without any further processing.

### 2.2. Corn Zein–Chitosan Composite Films

**Figure 1:**
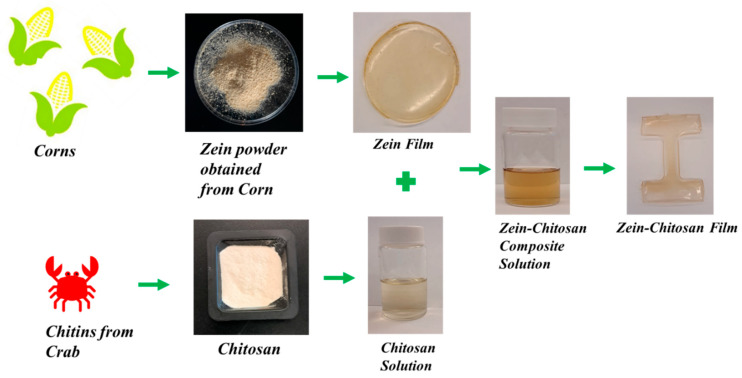
Synthesis of Corn Zein–Chitosan Composite Film. A two-step dissolution and blending were performed to achieve a more ordered molecular architecture within the composite blends, enhancing their properties.

Zein powder (10% *w*/*v*) was dissolved in formic acid and subsequently cast into films (Figure 1). After drying, the zein films were cut and dissolved in formic acid along with chitosan in selected weight-to-volume ratios to achieve a more ordered molecular architecture within the blend solution, and the resulting mixture was cast again to form composite films [19]. To remove any residual formic acid, the films were dried first in a fume hood and then in a vacuum oven at 60 °C overnight. The final films had an approximate thickness of 20 µm (Figure 1). The dried samples were analyzed both before and after NaOH (1% *w*/*v*) treatment at 25 ± 2 °C for 10 min, followed by rinsing with water.

### 2.3. Fourier Transform Infrared Spectroscopy (FTIR)

Fourier Transform Infrared (FTIR) spectroscopy was performed on untreated and NaOH-treated films, using a Bruker Tensor 27 instrument (Bruker, Billerica, MA, USA), which was fitted with a deuterated triglycine sulfate (DTGS) detector and operated under a constant nitrogen atmosphere to minimize atmospheric interference. Spectral data were acquired using an attenuated total reflectance (ATR) accessory with a Germanium crystal (MIRacle, Pike Technologies, Madison, WI, USA). The scans were recorded over a wavenumber range of 4000 to 400 cm^−1^, with 64 accumulations per sample at a resolution of 4 cm^−1^. To verify sample uniformity, spectra were recorded multiple times from both sides of each specimen. The ATR crystal was cleaned with methanol between measurements, and background scans (64 in total) were acquired before sample analysis.

To evaluate the degree of deacetylation (DD) in both untreated and NaOH-treated films, the original FTIR spectra was analyzed. The degree of acetylation (DA) was first calculated using the following equation [28]:

DA (%) = ((A_1320_/A_1420_) − 0.3822)/0.03133

where A_1320_ and A_1420_ represent the integrated absorbance areas of the bands at 1320 cm^−1^ and 1420 cm^−1^, respectively. The degree of deacetylation (DD) was then determined using the relation [29]:

DD (%) = 100% − DA (%)

### 2.4. Differential Scanning Calorimetry (DSC)

Thermal stability of the samples was evaluated using temperature-modulated differential scanning calorimetry (TM-DSC) on a TA Instruments DSC 2500 system (TA Instruments, New Castle, DE, USA) equipped with a refrigerated cooling system. Approximately 4–5 mg of each sample was sealed in aluminum pans for analysis. The temperature range for scanning was set from −40 °C to 400 °C, with a constant heating rate of 2 °C/min. Modulation parameters included a 60-s period and a temperature amplitude of 0.318 °C. A dry nitrogen atmosphere was maintained throughout the experiment with a flow rate of 50 mL/min. Before analysis, the instrument was calibrated for both temperature and heat flow using indium, and for heat capacity using aluminum and sapphire reference standards.

### 2.5. Thermal Gravitational Analysis (TGA)

Thermogravimetric analysis (TGA) was performed using a TA Instruments SDT-Q600 system (TA Instruments, New Castle, DE, USA) to monitor the weight changes in the film samples as a function of temperature. The measurements were conducted under a nitrogen atmosphere with a flow rate of 100 mL/min. Samples were heated from 25 °C to 800 °C at a constant rate of 10 °C/min, and the mass loss was continuously recorded throughout the temperature range.

### 2.6. Scanning Electron Microscopy (SEM)

Cross-sectional imaging was conducted on both untreated and NaOH-treated zein–chitosan blend films to evaluate their structural features across various composition ratios. A LEO 1530 VP scanning electron microscope (LEO Electron Microscopy Inc., Thornwood, NY, USA) was used for imaging, operating at an accelerating voltage of 10 kV with a magnification scale set to 5 µm. To prepare the samples, the films were first immersed in liquid nitrogen and then fractured to produce clean cross-sectional surfaces. The fractured pieces were mounted vertically on SEM stubs using double-sided carbon tape to ensure a 90° orientation relative to the stage. Images were captured from several regions along the cross-section of each film to assess structural consistency and homogeneity. Before imaging, a thin layer of gold was sputtered to the samples using a Denton Vacuum Desk II sputter coater (Denton Vacuum, Moorestown, NJ, USA) for 15 s to improve surface conductivity and image clarity.

## 3. Results and Discussion

### 3.1. Structural Analysis

**Figure 2:**
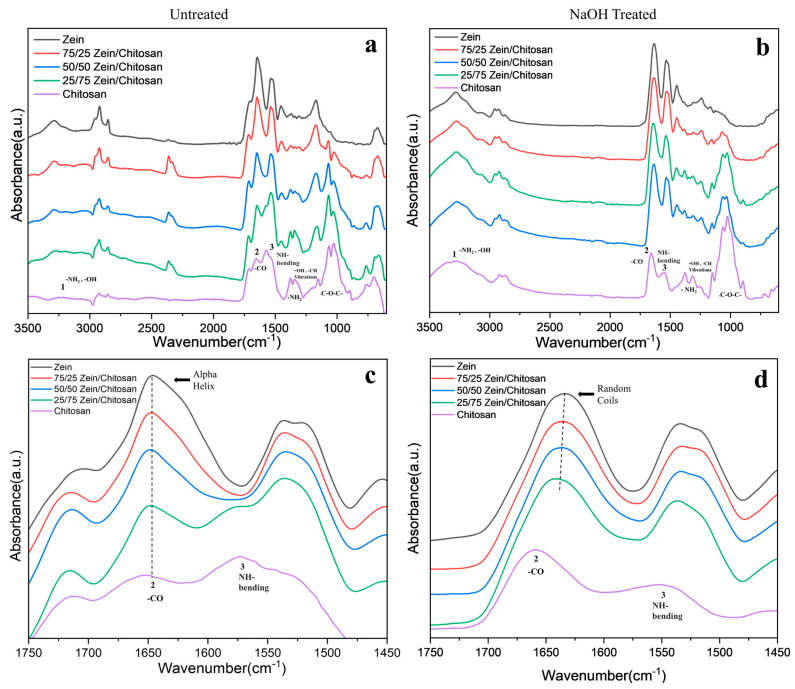
FTIR spectra of zein–chitosan composite films: (**a**,**c**) untreated and (**b**,**d**) NaOH-treated. (**c**,**d**) present zoomed-in views of the amide I and II regions.

FTIR analysis was performed on the samples to gain insight into the structural characteristics of the zein–chitosan composite films. In the untreated samples, pure zein exhibited a dominant α-helix conformation, as indicated by the absorption band near 1650 cm^−1^ (Figure 2a,c) [6,30]. However, following NaOH treatment and subsequent water wash, a clear conformational transition from α-helix to random coils was observed, as evidenced by a shift in the amide I band to approximately 1637 cm^−1^ as observed in Figure 2b,d. In parallel, the FTIR spectrum of the pure chitosan film exhibited distinct absorption bands at 1649 cm^−1^, 1572 cm^−1^, 1409 cm^−1^, 1150 cm^−1^, and 1060 cm^−1^, which are characteristic of its polysaccharide backbone (Figure 2a) [31,32,33]. Specifically, the band at 1650 cm^−1^ is attributed to C=O stretching, while the peak at 1578 cm^−1^ corresponds to –NH_2_ bending vibrations (Figure 2c). The absorption band observed at 1150 cm^−1^ is associated with the asymmetric stretching of the C–O–C bridge, and the bands near 1060 cm^−1^ and 1026 cm^−1^ arise from skeletal vibrations involving C–O stretching modes (Figure 2a) [18]. These spectral features further confirm the presence of residual acetyl groups, indicative of partial deacetylation of chitin [19]. Whereas the band at 1320 cm^−1^ corresponds to the C-N stretching of amide III, the band at 1420 cm^−1^ refers to the vibrations of C–H and O–H groups in the ring [16]. After NaOH treatment, the observed shifts in bands 1, 2, and 3 (Figure 2a,b) in the pure chitosan film from 3213 to 3278 cm^−1^, 1649 to 1653 cm^−1^, and 1572 to 1542 cm^−1^, which can be attributed to the deprotonation of amine groups upon exposure to sodium hydroxide. This loss of protons led to a reduction in the hydration shell surrounding the amine groups, thereby facilitating the formation of new hydrogen bonds within the chitosan chains [16]. As the chitosan content in the blend films decreased, a corresponding reduction in the intensity of the C–O stretching absorption bands at 1059 and 1026 cm^−1^ was observed in composite films for both untreated and NaOH-treated samples (Figure 2a,b) [18]. In addition, a noticeable shift in the amide I band toward higher wavenumbers occurred with increasing zein content (Figure 2c,d). This shift, present in both treated and untreated films, suggests the formation of hydrogen bonds between chitosan and zein, indicating molecular interactions within the blended structure. Moreover, NaOH treatment increased the deacetylation of chitosan in the composites (Table 1), collectively enhancing molecular interactions and resulting in a stable network [16]. These peak shifts and spectral changes not only reflect the molecular-level interactions but also highlight the significance of hydrogen bonding interactions in both untreated and treated films, providing valuable insight into the structural evolution of these hybrid systems. Findings from FTIR illustrate how variations in composition and processing conditions can be employed to strategically tailor the structural and thermal properties of the films. This tunability opens promising opportunities for their application in high-performance composite systems.

### 3.2. Thermogravimetric Analysis

**Figure 3:**
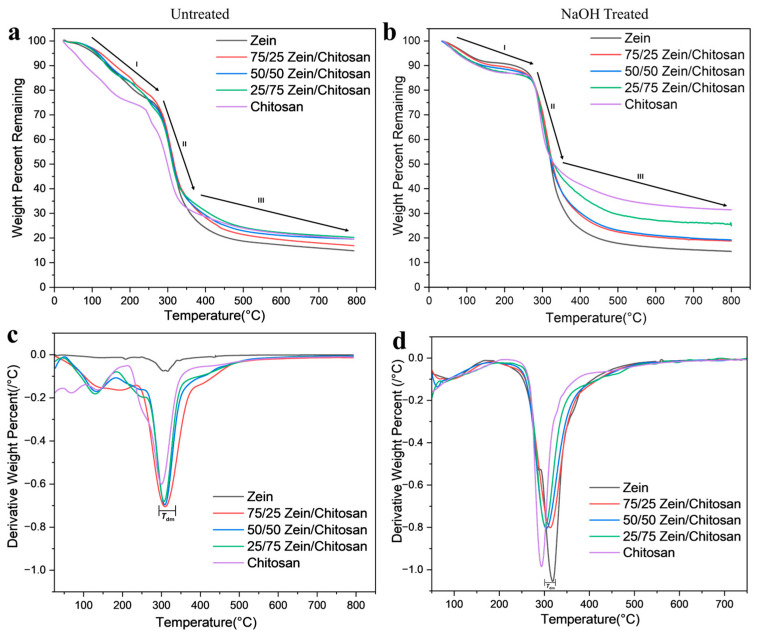
Thermogravimetric analysis of zein–chitosan composite films: (**a**,**c**) untreated and (**b**,**d**) NaOH-treated. (**c**,**d**) present derivative of remaining weight percentage, as the same samples were heated up to 800 °C.

Thermogravimetric analysis (TGA) was carried out to assess the thermal stability of zein–chitosan films, including both untreated and NaOH-treated samples. The thermograms presented in Figure 3 reveal that all samples experienced a slight initial weight loss below 200 °C (Region I), primarily due to the evaporation of residual moisture and loosely bound solvents. This was followed by a rapid degradation phase between 250 °C and 325 °C (Region II), and then a steadier decomposition stage beyond 350 °C (Region III). By 800 °C, most samples exhibited a total mass loss of approximately 40–50%.

Among the films analyzed for untreated samples (Figure 3a), pure chitosan exhibited high thermal stability at 800 °C, maintaining its structure at elevated temperatures, while pure zein degraded more rapidly above 300 °C. Blended films with higher chitosan content demonstrated increased thermal resistance, as evidenced by elevated onset decomposition temperatures and higher degradation midpoint temperatures (*T*_dm_), reflected by the derivative peak in Figure 3c. The relatively low thermal stability of pure zein may be attributed to its hydrophobic character. Notably, composites containing 75% chitosan showed a significant upward shift in *T*_dm_ (Figure 3c), suggesting that chitosan plays a dominant role in enhancing the overall thermal performance of the blend. After NaOH treatment, the blends showed a significant improvement in thermal stability (Figure 3b). The weight loss of pure chitosan improved from 19.58% to 31.40% at 800 °C. Similarly, chitosan-dominant samples, such as 25/75 zein–chitosan and 50/50 zein–chitosan, also exhibited a marked increase in thermal stability. This enhancement can be attributed to the formation of new hydrogen bonds and increased intermolecular interactions between the side chains of zein and chitosan, as evidenced by FTIR analysis. In contrast, the thermal stability of pure zein and zein-dominant blends remained largely unchanged, likely because zein’s globular structure and hydrophobic interactions are less responsive to alkaline treatment further indicating the structural stability of zein at any conditions. The derivative of weight percent (Figure 3d) shows that pure chitosan has a lower *T*_dm_ compared to the composites, while pure zein exhibits the highest *T*_dm_. The composites lie in between, indicating that blending enhances chitosan’s thermal stability while slightly affecting zein, resulting in intermediate degradation behavior.

### 3.3. Differential Scanning Calorimetry

**Figure 4:**
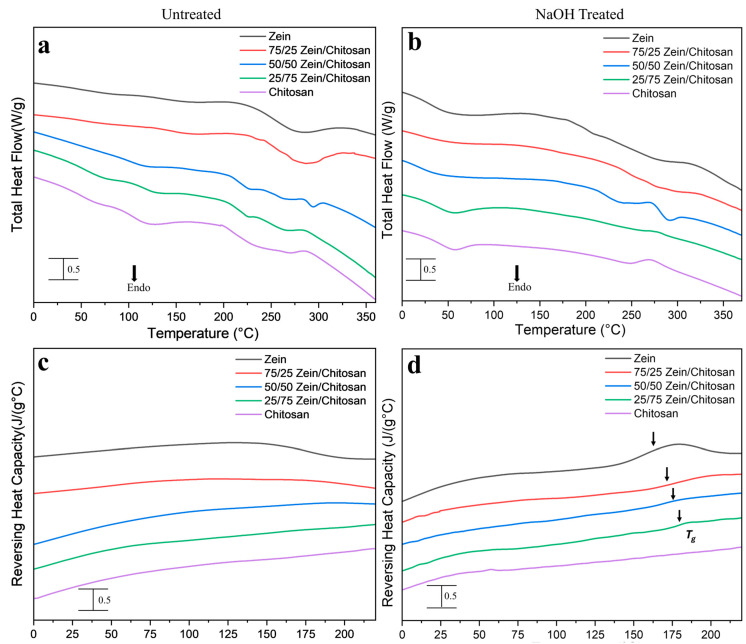
DSC thermograms of zein–chitosan films before and after NaOH treatment. (**a**,**b**) represent total heat flow, while (**c**,**d**) show reversing heat capacity, reflecting thermal and structural transitions. Arrow shows the position of each Tg.

Differential Scanning Calorimetry (DSC) was utilized to investigate the thermal transitions of zein–chitosan films before and after treatment (Figure 4). Figure 4a shows the total heat flow profile for the untreated samples. All film compositions exhibited endothermic peaks below 150 °C, which corresponded to the release of bound water absorbed from the environment. Following the removal of water, a gradual decline in heat flow was evident with increasing temperature, reflecting the enhanced molecular mobility of the protein chains as temperature increases. In the case of pure zein films, a distinct exothermic transition was observed at approximately 210 °C (Figure 4a). This thermal event is consistent with literature reports and is attributed to the reorganization of zein’s molecular structure, specifically the transition toward β-structures [9,34,35]. In addition to pure zein, zein-dominant composite films exhibited exothermic transitions just prior to the onset of thermal degradation, typically characterized by a single-step decomposition process [9]. A prominent endothermic transition observed at 284.6 °C in the pure zein film (Figure 4a) corresponds to its thermal degradation [6]. This thermal degradation peak shifted to approximately 286.7 °C and 297.0 °C with the incorporation of 25% and 50% chitosan, respectively. In contrast, the pure chitosan film exhibited a distinct exothermic peak at 288.1 °C. This exothermic peak present in chitosan can be attributed to dehydration of the saccharide rings, depolymerization, and the breakdown of both acetylated and deacetylated segments of the polymer chain [18,36,37,38]. Notably, the DSC thermograms of the zein–chitosan composite with a 25/75 Zein/chitosan ratio exhibited an exothermic peak closely resembling that of pure chitosan. In contrast, the 50/50 and 75/25 zein–chitosan blends displayed endothermic peaks characteristic of zein. As the chitosan content increased, the thermal degradation temperature of the zein component gradually shifted to higher values. This upward shift suggests enhanced thermal stability, likely resulting from the formation of intermolecular interactions between zein and chitosan within the composite matrix. Figure 4c highlights the glass transition temperatures (*T*_g_) of the zein–chitosan composite films based on the reversing heat capacity curves. However, the *T*_g_ values of the untreated pure zein, chitosan and their composite samples are not clearly distinguishable, likely due to overlapping thermal events or weak transitions that fall below the instrument’s sensitivity. This may result from the semi-crystalline nature of chitosan and the plasticizing effect of residual solvents in zein, both of which can obscure or broaden the *T*_g_ signal.

After NaOH treatment (Figure 4b), the glass transition temperatures (*T*_g_) of pure zein and its composites became more distinguishable. Specifically, pure zein exhibited a *T*_g_ at 172.5 °C, whereas the composite films showed a gradual increase in *T*_g_ with higher chitosan content: 175.1 °C for the 75/25 zein/chitosan ratio, 176.2 °C for 50/50, and 177.8 °C for 25/75, as shown in Figure 4d. In addition, a solvent evaporation endotherm was observed in the range of 45–57 °C, which progressively shifted from zein to chitosan compositions, indicating compositional influence on solvent retention. Furthermore, thermal degradation peaks were recorded as follows: pure zein degraded at approximately 281.3 °C; the 75/25 zein/chitosan composite showed a peak at 288.4 °C; the 50/50 composite at 292.4 °C; the 25/75 zein/chitosan composite exhibited an exothermic peak at 277.1 °C; and pure chitosan showed a degradation peak around 271.6 °C after NaOH treatment. The altered thermal events observed in chitosan and its composites, compared to the untreated sample, may be attributed to the reduction in the hydration shell surrounding the amine groups and the thermal degradation of zein’s random coil structures.

### 3.4. Scanning Electron Microscopy

**Figure 5:**
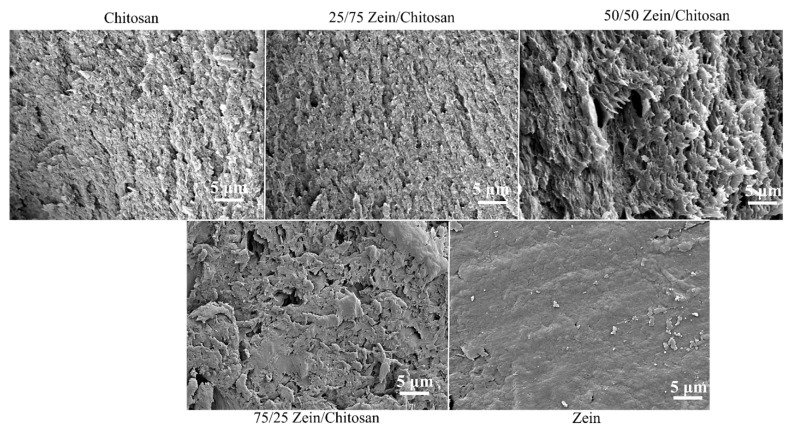
SEM images of zein–chitosan composite films before treatment, showing surface morphology across different blend ratios.

The cross-sections of untreated zein–chitosan composite films exhibited distinct and consistent morphological features across varying compositions, as observed by scanning electron microscopy (SEM) and presented in Figure 5. Pure zein films appeared smooth but brittle, whereas pure chitosan films were thin, flexible, and mechanically robust, likely due to their more ordered and cohesive molecular architecture at the micro/nano scale. The morphological properties of the composite films were found to be tunable by adjusting the zein-to-chitosan ratio. Notably, untreated chitosan-dominant films (25/75 and 50/50) exhibited pronounced surface nanofibrillar ridges resembling those of pure chitosan as observed in previous works [19], whereas zein-rich films (75/25) displayed irregular and disordered surface topographies, diverging from smooth features characteristic of pure zein.

**Figure 6:**
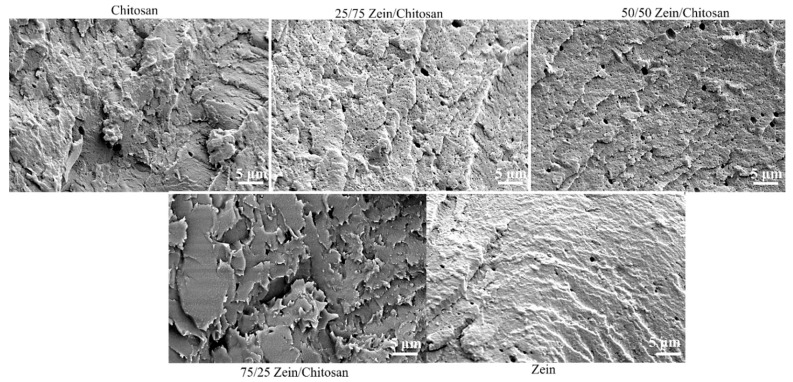
SEM images of zein–chitosan composite films after NaOH treatment, showing surface morphology across different blend ratios.

To further elucidate the morphological evolution at the microscale, the cross-sections of NaOH-treated composite films were also analyzed by SEM, as presented in Figure 6. Comparisons with the untreated pure components (Figure 5) revealed significant changes in surface features upon composites. The pure chitosan film exhibited a continuous and homogeneous surface with rough, aligned ridging, while the pure NaOH-treated zein displayed a rougher texture. All composite films demonstrated an overall increase in surface roughness at the microscopic level.

An increase in chitosan content generally introduced a more porous and rougher surface morphology. Both the 50/50 and 75/25 compositions exhibited intermittent smooth domains interspersed with rough, porous regions. In the 75/25 zein–chitosan blend, more defined surface ridges with occasional microcracks were observed, stress-induced micro fracturing which might be due to alkaline treatment. These morphological transitions after NaOH treatment from smooth and featureless to rough and porous suggest that zein–chitosan composites possess tunable surface properties with promising applications in material and biological adhesion.

### 3.5. Mechanism of Zein–Chitosan Interaction

**Figure 7:**
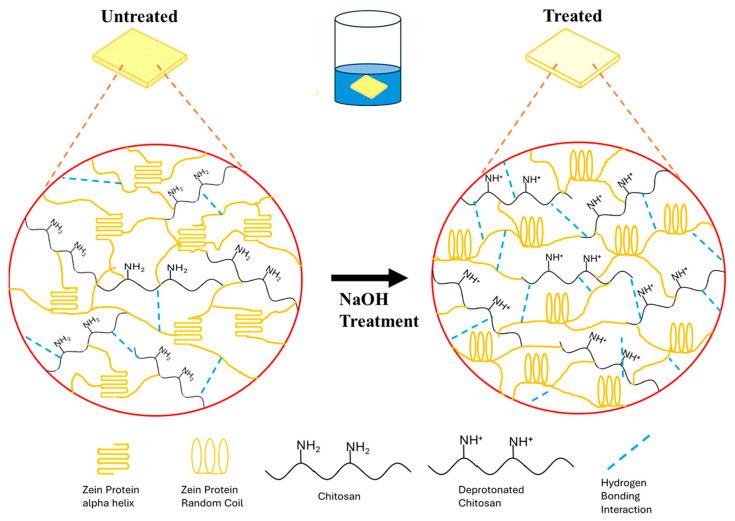
Schematic illustration of the protein-polysaccharide interactions in zein–chitosan composite films before and after NaOH treatment.

The structural evolution of zein–chitosan composite films can be interpreted through structural, thermal and morphological analyses. Figure 7 presents a schematic illustration of the protein–polysaccharide interactions in zein–chitosan composite films before and after NaOH treatment. In untreated zein films, the protein exhibits a high content of α-helical structures, as indicated by the Amide I peak around 1650 cm^−1^. After NaOH treatment, a shift in the Amide I bands toward ~1640 cm^−1^ suggests a transition from α-helical structures to random coil conformations, likely due to partial unfolding of zein’s polypeptide chains. In the case of chitosan, NaOH treatment leads to the deprotonation of its amine groups, reducing the hydration shell around the –NH^+^ groups. This facilitates the formation of stronger hydrogen bonds within and between polymer chains, as evidenced by shifts in bands I, II, and III in the FTIR spectra. These structural reorganizations enhance the rigidity and internal cohesion of the chitosan matrix.

When blended, zein and chitosan form composite films through a combination of electrostatic interactions, hydrogen bonding, and hydrophobic associations. NaOH treatment further enhances the compatibility and interaction between the two biopolymers by promoting structural reorganization and zein becomes more flexible due to reduced helix structure, while chitosan gains conformational stability. As a result, the composite films exhibit improved thermal stability and more uniform morphology at the micro/nano scale, making them suitable for applications in food packaging, drug delivery, and adhesive biomaterials.

## 4. Conclusions

This study demonstrates that controlled alkaline treatment is an effective strategy for modulating the structural and physicochemical properties of zein–chitosan composite films. By combining zein, a hydrophobic plant-derived protein, with chitosan, a cationic polysaccharide, the resulting composites leverage the complementary characteristics of both biopolymers to achieve tunable material performance. Comprehensive spectroscopic and thermal analyses revealed that NaOH treatment induces deprotonation of chitosan’s amine groups, increases its degree of deacetylation, and partially unfolds zein’s α-helical structures, thereby enhancing hydrogen bonding and electrostatic interactions. These molecular-level modifications lead to the formation of a denser and more organized composite network, as further evidenced by SEM, which showed improved surface uniformity and the development of porous microstructures. Importantly, the post-synthetic alkaline treatment offers a simple, scalable, and effective means of tailoring material properties without the need for complex chemical modifications. This work provides valuable insight into the hierarchical assembly and functional tuning of protein–polysaccharide composites and supports the continued development of zein–chitosan systems as sustainable, biodegradable platforms for advanced biomedical and packaging applications.

## Figures and Tables

**Table 1:** Degree of deacetylation (DD) of chitosan and its composites, both untreated and NaOH-treated, as determined by FTIR spectral analysis.

Film Composition	Untreated (%, DD)	NaOH Treated (%, DD)
Chitosan	74.21	84.08
75/25 Chitosan/Zein	70.63	73.44
50/50 Chitosan/Zein	69.36	70.58
25/75 Chitosan/Zein	60.56	67.02

## Data Availability

The original contributions presented in this study are included in the article. Further inquiries can be directed to the corresponding author.

## References

[1] Hu X., Cebe P., Weiss A.S., Omenetto F., Kaplan D.L. (2012). Protein-based composite materials. Mater. Today.

[2] Hu X., Duki S., Forys J., Hettinger J., Buchicchio J., Dobbins T., Yang C. (2014). Designing silk-silk protein alloy materials for biomedical applications. J. Vis. Exp..

[3] Zhang Y., Cui L., Che X., Zhang H., Shi N., Li C., Chen Y., Kong W. (2015). Zein-based films and their usage for controlled delivery: Origin, classes and current landscape. J. Control. Release.

[4] Argos P., Pedersen K., Marks M.D., Larkins B.A.A. (1982). structural model for maize zein proteins. J. Biol. Chem..

[5] Matsushima N., Danno G.-I., Takezawa H., Izumi Y. (1997). Three-dimensional structure of maize α-zein proteins studied by small-angle X-ray scattering. Biochim. Biophys. Acta (BBA)-Protein Struct. Mol. Enzym..

[6] Poluri N., Gough C.R., Sanderlin S., Velardo C., Barca A., Pinto J., Perrotta J., Cohen M., Hu X. (2025). Silk-Corn Zein Alloy Materials: Influence of Silk Types (Mori, Thai, Muga, Tussah, and Eri) on the Structure, Properties, and Functionality of Insect—Plant Protein Blends. Int. J. Mol. Sci..

[7] Reboredo C., González-Navarro C.J., Martínez-López A.L., Martínez-Ohárriz C., Sarmento B., Irache J.M. (2021). Zein-Based Nanoparticles as Oral Carriers for Insulin Delivery. Pharmaceutics.

[8] Chen H., Su J., Brennan C.S., Van der Meeren P., Zhang N., Tong Y., Wang P. (2022). Recent developments of electrospun zein nanofibres: Strategies, fabrication and therapeutic applications Recent developments of electrospun zein nanofibres: Strategies, fabrication and therapeutic applications. Mater. Today Adv..

[9] DeFrates K., Markiewicz T., Xue Y., Callaway K., Gough C., Moore R., Bessette K., Mou X., Hu X. (2021). Air-jet spinning corn zein protein nanofibers for drug delivery: Effect of biomaterial structure and shape on release properties. Mater. Sci. Eng. C Mater. Biol. Appl..

[10] Mouzakitis C.K., Sereti V., Matsakidou A., Kotsiou K., Biliaderis C.G., Lazaridou A. (2022). Physicochemical properties of zein-based edible films and coatings for extending wheat bread shelf life. Food Hydrocoll..

[11] Corradini E., Souto De Medeiros E., Carvalho A.J.F., Curvelo A.A.S., Mattoso L.H.C. (2006). Mechanical and morphological characterization of starch/zein blends plasticized with glycerol. J. Appl. Polym. Sci..

[12] Huang W., Zou T., Li S., Jing J., Xia X., Liu X. (2013). Drug-Loaded Zein Nanofibers Prepared Using a Modified Coaxial Electrospinning Process. AAPS PharmSciTech.

[13] Yao C., Li X., Song T., Li Y., Pu Y. (2009). Biodegradable nanofibrous membrane of zein/silk fibroin by electrospinning. Polym. Int..

[14] Aranaz I., Alcántara A.R., Civera M.C., Arias C., Elorza B., Heras Caballero A., Acosta N. (2021). Chitosan: An Overview of Its Properties and Applications. Polymers.

[15] Mittal H., Ray S.S., Kaith B.S., Bhatia J.K., Sukriti, Sharma J., Alhassan S.M. (2018). Recent progress in the structural modification of chitosan for applications in diversified biomedical fields. Eur. Polym. J..

[16] Takara E.A., Marchese J., Ochoa N.A. (2015). NaOH treatment of chitosan films: Impact on macromolecular structure and film properties. Carbohydr. Polym..

[17] Schmuhl R., Krieg H.M., Keizer K. (2001). Adsorption of Cu (II) and Cr (VI) ions by chitosan: Kinetics and equilibrium studies. Water SA.

[18] Moraes M.A.d., Nogueira G.M., Weska R.F., Beppu M.M. (2010). Preparation and Characterization of Insoluble Silk Fibroin/Chitosan Blend Films. Polymers.

[19] Huang J., Qin J., Zhang P., Chen X., You X., Zhang F., Zuo B., Yao M. (2020). Facile preparation of a strong chitosan-silk biocomposite film. Carbohydr. Polym..

[20] Li B., Qiu L., Zhang J., Liu S., Xu M., Wang J., Yang H. (2023). Solubilization of chitosan in biologically relevant solvents by a low-temperature solvent-exchange method for developing biocompatible chitosan materials. Int. J. Biol. Macromol..

[21] Sakai Y., Hayano K., Yoshioka H., Yoshioka H. (2001). A Novel Method of Dissolving Chitosan in Water for Industrial Application. Polym. J..

[22] Sharkawy A., Barreiro M.F., Rodrigues A.E. (2020). Chitosan-based Pickering emulsions and their applications: A review. Carbohydr. Polym..

[23] Loryuenyong V., Nakhlo W., Srikaenkaew P., Yaidee P., Eiad-Ua A., Buasri A. (2025). Optimization and performance prediction of carbon dioxide adsorption on chitosan/ activated carbon/epichlorohydrin composite materials using Box–Behnken design and artificial neural network approaches. Case Stud. Chem. Environ. Eng..

[24] Oktay B., Ciftci F., Erarslan A., Ahlatcıoğlu Özerol E. (2025). Dual-layer Natamycin and Boric-Acid-reinforced PVA/chitosan by 3D printing and electrospinning method: Characterization and In Vitro evaluation. Polymers.

[25] Brito G.B., Pinho-Jr J.d.S., Guimarães A.d.S., Conte-Júnior C.A., Nele M., Perrone D., Castelo-Branco V.N. (2025). Optimization and characterization of crosslinked chitosan-based oleogels based on mechanical properties of conventional solid fats. Polymers.

[26] Yu C., Sun H., Yao L., Weng Y. (2025). NaOH/Urea-Compatible Chitosan/Carboxymethylcellulose Films: Orthogonal Optimization of Packaging Properties. Molecules.

[27] Masanabo M.A., Ray S.S., Emmambux M.N. (2022). Properties of thermoplastic maize starch-zein composite films prepared by extrusion process under alkaline conditions. Int. J. Biol. Macromol..

[28] Czechowska-Biskup R., Jarosinska D., Rokita B., Ulanski P., Rosiak J.M. (2012). Determination of degree of deacetylation of chitosan-comparison of methods. Prog. Chem. Appl. Chitin Deriv..

[29] Sánchez-Machado D.I., López-Cervantes J., Escárcega-Galaz A.A., Campas-Baypoli O.N., Martínez-Ibarra D.M., Rascón-León S. (2024). Measurement of the degree of deacetylation in chitosan films by FTIR, 1H NMR and UV spectrophotometry. MethodsX.

[30] Gillgren T., Barker S.A., Belton P.S., Georget D.M.R., Stading M. (2009). Plasticization of Zein: A Thermomechanical, FTIR, and Dielectric Study. Biomacromolecules.

[31] Gu Z., Xie H., Huang C., Li L., Yu X. (2013). Preparation of chitosan/silk fibroin blending membrane fixed with alginate dialdehyde for wound dressing. Int. J. Biol. Macromol..

[32] Fernandez J.G., Ingber D.E. (2012). Unexpected Strength and Toughness in Chitosan-Fibroin Laminates Inspired by Insect Cuticle. Adv. Mater..

[33] Li Z., Jiang X., Huang H., Liu A., Liu H., Abid N., Ming L. (2022). Chitosan/zein films incorporated with essential oil nanoparticles and nanoemulsions: Similarities and differences. Int. J. Biol. Macromol..

[34] Magoshi J., Nakamura S., Murakami K.I. (1992). Structure and physical properties of seed proteins. I. Glass transition and crystallization of zein protein from corn. J. Appl. Polym. Sci..

[35] Shukla R., Cheryan M. (2001). Zein: The industrial protein from corn. Ind. Crops Prod..

[36] Kweon H.Y., Um I.C., Park Y.H. (2001). Structural and thermal characteristics of *Antheraea pernyi* silk fibroin/chitosan blend film. Polymer.

[37] de Farias B.S., Grundmann D.D.R., Rizzi F.Z., Martins N.S.S., Sant’Anna Cadaval Junior T.R., de Almeida Pinto L.A. (2019). Production of low molecular weight chitosan by acid and oxidative pathways: Effect on physicochemical properties. Food Res. Int..

[38] Kumar S., Dutta J., Dutta P.K. (2009). Preparation and characterization of N-heterocyclic chitosan derivative based gels for biomedical applications. Int. J. Biol. Macromol..

